# Less sitting, more physical activity and higher cardiorespiratory fitness: associations with weight status among a national sample of children

**DOI:** 10.15171/hpp.2017.31

**Published:** 2017-06-14

**Authors:** Paul D. Loprinzi, Meghan K. Edwards

**Affiliations:** ^1^Jackson Heart Study Vanguard Center of Oxford, Physical Activity Epidemiology Laboratory, Exercise Psychology Laboratory, Department of Health, Exercise Science and Recreation Management, The University of Mississippi, University, MS 38677, USA; ^2^Physical Activity Epidemiology Laboratory, Exercise Psychology Laboratory, Department of Health, Exercise Science and Recreation Management, The University of Mississippi, University, MS 38677, USA

**Keywords:** Fitness, NHANES, Obesity, Youth

## Abstract

**Background:** Very few studies have evaluated the independent and combined associations of sedentary behavior (SB), moderate-to-vigorous physical activity (MVPA) and cardiorespiratory fitness (CRF) on obesity. Our recent work has evaluated this paradigm in the adult population,but no study has evaluated this paradigm in the child population, which was the purpose of this study.

**Methods:** A national sample of children (N=680, 6-11 years) were evaluated via the National Youth Fitness Survey; this study was conducted in 2012, employing a nationally representative sample, occurring across 15 different geographic regions in the United States. SB and MVPA were assessed via parental recall, with CRF objectively measured via a treadmill-based aerobic test. Obesity was determined for measured body mass index. A PACS (Physical Activity Cardiorespiratory Sedentary) score was created ranging from 0-3, indicating each child’s number of positive characteristics (PA, CRF, SB).

**Results: ** Meeting MVPA guidelines (OR _adjusted_=0.47; 95% CI: 0.29-0.77) and above-median CRF (OR _adjusted_=0.12; 95% CI: 0.07-0.21), but not SB (OR _adjusted_=0.62; 95% CI: 0.35-1.10),were associated with reduced odds of obesity. Compared to those with a PACS score of 0, the odds of obesity for PACS scores of 1-3, respectively, were: 0.31 (0.18-0.53), 0.12 (0.04-0.34), and 0.05 (0.02-0.10).

**Conclusion:** These findings highlight the need for public health strategies to promote child MVPA and CRF, and to reduce SB.

## Introduction

Recent declines in youth physical activity (PA) levels^1^ are concerning, given irrefutable evidence that regular PA has myriad health benefits among this population.^2,3^ While regular PA among children may enhance cardiorespiratory fitness (CRF),^2^ CRF has been shown to associate with health,^4^ independent of PA,^4^ underscoring the importance of evaluating the unique health effects of PA and CRF. Further, though not conclusive within the adult population, prolonged sedentary behavior (SB) unfavorably associates with health outcomes among adults and children.^5^ Notably, few studies^4^ have evaluated the independent and cumulative associations of PA, CRF, and SB with health, and no studies have examined this paradigm among children, which was the purpose of this *short communication*. Within a national sample of children, we examined the independent and cumulative associations of PA, CRF and SB on weight status. This investigation is of importance given the recent trends,^6^ high prevalence (17.7%),^6^ and deleterious consequences associated with childhood obesity.^2,3^

## Materials and Methods

### Study design & participants

The present study employed the National Youth Fitness Survey (NYFS), conducted in 2012 by the Division of Health and Nutrition Examination Surveys, National Center for Health Statistics, as part of the Centers for Disease Control and Prevention. The NYFS utilizes a multistage probability design. The unweighted analytic sample included 680 children (6-11 years). The weighted sample included 22.8 million children (in the United States).

### Physical activity

Parents were asked, “*During the past 7 days, on how many days was your child physically active for a total of at least 60 minutes per day*?” Meeting PA guidelines was defined as ≥60 min/d of PA for all 7 days.

### Sedentary behavior

Parents were asked, “*Over the past 30 days, on average how many hours per day did your child sit and watch TV, videos or play computer games*?” Elevated SB was defined as > 2 h/d of SB.

### Cardiorespiratory fitness

The 2012 NYFS provides the first nationally representative, objectively measured (via treadmill-based testing) CRF data among children. Using age-specific maximal exercise treadmill protocols, children exercised to the point of volitional fatigue, with endurance time (s) as the outcome measure. High CRF was defined as >660 seconds (the sample median) endurance time.

### Weight status

Weight status was derived using measured body mass index (BMI) percentiles from the sex-specific 2000 BMI-for-age CDC growth curves.

### Analysis

Weighted multivariable logistic regression analyses examined the independent and cumulative associations of PA, CRF and SB with obesity (Stata version12; College Station, TX, USA). A PACS^4^ (Physical Activity Cardiorespiratory Sedentary) score was created ranging from 0-3, indicating each child’s number of positive characteristics (PA, CRF, SB). Covariates included age, gender, race-ethnicity and asthma status. There was no collinearity in the model; the highest variance inflation factor was 1.2, the mean variance inflation factor was 1.0, the lowest tolerance statistic was 0.84, and the highest correlation between any two parameters in the model was r = 0.38. Statistical significance was established as *P *< 0.05.

## Results

The mean age was 8.4 years (SD 0.04); 50.3% boys; 58.2% met PA guidelines; 46.4% did not have elevated SB; and the respective number of 0, 1, 2, and 3 PACS scores were 107, 196, 227, and 150.

Meeting PA guidelines (OR_adjusted _= 0.47; 95% CI: 0.29-0.77) and above-median CRF (OR_adjusted _= 0.12; 95% CI: 0.07-0.21), but not SB (OR_adjusted _= 0.62; 95% CI: 0.35-1.10), were associated with reduced odds of obesity. Compared to those with a PACS score of 0, the odds of obesity for PACS scores of 1-3, respectively, were: 0.31 (0.18-0.53), 0.12 (0.04-0.34), and 0.05 (0.02-0.10).

## Discussion

The present study employed a national sample of children to evaluate the potential independent and combined associations of PA, SB, and CRF on obesity. The motivation for this study was our previous work on this topic among adults, but to date, this specific question has not been evaluated extensively in the child population. Corroborating findings in an adult sample,^4^ in this national sample of children, PA and CRF, but not SB, were associated with reduced odds of obesity. Those with a PACS score of 3 had the lowest odds of being obese.

The findings that those with a PACS score of 3 had the lowest odds of being obese highlights the need for public health strategies to promote child PA and CRF, and to reduce SB. One such strategy for children may be to make modifications to school-based activities (e.g., integrating movement into curriculum) as youth tend to engage in longer SB bouts during school when compared to non-school hours.^2,5,7^ Other sensible strategies include the integration of effective, daily physical education courses during the school hours.^2^ Despite the notable strengths of this study, which include the novel topic and national sample of children, a limitation of this study is the cross-sectional design, rendering causality not possible. Thus, prospective work on this novel paradigm is warranted. Confirmation of these findings by future work will help shed light on identifying effective movement-based strategies to help prevent and treat obesity among children.

## Ethical Approval

This study was approved by the ethics committee at the National Center for Health Statistics.

## Competing interests

We declare no conflicts of interest.

## Authors’ contributions

All authors were involved in the conceptualization of the study, revising the manuscript and interpreting the results. PDL computed the analyses and drafted the first draft of the manuscript.

## Acknowledgments

No funding was used to prepare this manuscript.
